# Monitoring Mentions of COVID-19 Vaccine Side Effects on Japanese and Indonesian Twitter: Infodemiological Study

**DOI:** 10.2196/39504

**Published:** 2022-10-04

**Authors:** Kiki Ferawati, Kongmeng Liew, Eiji Aramaki, Shoko Wakamiya

**Affiliations:** 1 Graduate School of Science and Technology Nara Institute of Science and Technology Ikoma Japan

**Keywords:** COVID-19, vaccine, COVID-19 vaccine, Pfizer, Moderna, vaccine side effects, side effects, Twitter, logistic regression

## Abstract

**Background:**

The year 2021 was marked by vaccinations against COVID-19, which spurred wider discussion among the general population, with some in favor and some against vaccination. Twitter, a popular social media platform, was instrumental in providing information about the COVID-19 vaccine and has been effective in observing public reactions. We focused on tweets from Japan and Indonesia, 2 countries with a large Twitter-using population, where concerns about side effects were consistently stated as a strong reason for vaccine hesitancy.

**Objective:**

This study aimed to investigate how Twitter was used to report vaccine-related side effects and to compare the mentions of these side effects from 2 messenger RNA (mRNA) vaccine types developed by Pfizer and Moderna, in Japan and Indonesia.

**Methods:**

We obtained tweet data from Twitter using Japanese and Indonesian keywords related to COVID-19 vaccines and their side effects from January 1, 2021, to December 31, 2021. We then removed users with a high frequency of tweets and merged the tweets from multiple users as a single sentence to focus on user-level analysis, resulting in a total of 214,165 users (Japan) and 12,289 users (Indonesia). Then, we filtered the data to select tweets mentioning Pfizer or Moderna only and removed tweets mentioning both. We compared the side effect counts to the public reports released by Pfizer and Moderna. Afterward, logistic regression models were used to compare the side effects for the Pfizer and Moderna vaccines for each country.

**Results:**

We observed some differences in the ratio of side effects between the public reports and tweets. Specifically, fever was mentioned much more frequently in tweets than would be expected based on the public reports. We also observed differences in side effects reported between Pfizer and Moderna vaccines from Japan and Indonesia, with more side effects reported for the Pfizer vaccine in Japanese tweets and more side effects with the Moderna vaccine reported in Indonesian tweets.

**Conclusions:**

We note the possible consequences of vaccine side effect surveillance on Twitter and information dissemination, in that fever appears to be over-represented. This could be due to fever possibly having a higher severity or measurability, and further implications are discussed.

## Introduction

### Background

Vaccinations have been proposed as one of the solutions to contain and end the COVID-19 pandemic [1-3]. Prior to their widespread deployment, an early Twitter poll suggested that public sentiment toward vaccinations was mostly positive, with many individuals indicating that they would seek vaccination, despite ongoing concerns about the safety of the vaccines [4]. However, these concerns persisted in the public eye, including issues like safety, the unusually quick development of vaccines, and possible side effects after administration [5]. These were also observed in vaccine-related search trends in early 2021, when resulting side effects were identified as a significant area of concern [6].

The vaccine rollout evoked a diverse set of reactions from the general population, be it for or against vaccination. Bonnevie et al [7] investigated vaccine acceptance before the pandemic and in the middle of pandemic in 2020 and found stronger vaccine opposition on Twitter during the latter period. The trend appeared reversed in the study conducted by Lyu et al [8], who studied public perception and reactions toward COVID-19 vaccinations on Twitter through topic modeling and sentiment analysis and found that early discussions about vaccines stemmed from the development stage of the vaccines, and public sentiment leaned toward a positive outlook later on.

As one of the most popular social media platforms in use today, Twitter has been widely utilized as a source for research in COVID-19 infodemiology (see [9]), building on an extant body of literature on epidemic surveillance via that platform. For example, in the case of influenza, Twitter has been used as a detection tool to estimate individual diagnoses [10] and as social surveillance, functioning as an early warning tool for outbreak detection [11]. Despite the usefulness of Twitter for epidemiological surveillance studies, there are limitations, such as the spread of misinformation during the early periods of the COVID-19 pandemic [12] that may bias these data. In this paper, we proposed that Twitter can also be used in a similar fashion to monitor side effects from COVID-19 vaccination, by focusing on the Pfizer and Moderna vaccines in Japan and Indonesia.

### Attitudes Toward COVID-19 Vaccination

Most studies on COVID-19 vaccination and Twitter have focused on general collective attitudes toward vaccination. Marcec and Likic [13] applied lexicon-based sentiment analysis to English tweets mentioning AstraZeneca, Pfizer, and Moderna and found that the sentiment for Pfizer and Moderna was generally more positive than that for AstraZeneca. Sattar and Arifuzzaman [14] analyzed tweets related to public sentiment about COVID-19 vaccination awareness and found strong positive sentiments despite the side effects of the vaccine. Kwok et al [15] used latent Dirichlet allocation topic modeling to identify topics in tweets related to COVID-19 vaccination in Australia, applied sentiment analysis to the tweets, and found that counts of tweets with positive sentiment were only slightly larger than counts of tweets with negative sentiment, thereby raising concerns over widespread vaccine acceptance. Yet, most of these studies were with Western and English samples, and there has been a considerable lack of similar research for tweets in non-English languages.

In this paper, we focused on tweets concerning widely available messenger RNA (mRNA) vaccines in Japan and Indonesia, 2 island countries located in the Pacific that have a large Twitter-using population (top 10 in terms of Twitter users globally [16]). As the Japanese and Indonesian languages are largely ubiquitously spoken in their respective countries [17,18], it provides for a relatively controlled environment to observe patterns unique to each country. This allows for a contextualization of Twitter usage to the wider society for added interpretations and behavioral analyses, especially in this pandemic.

The year 2021 saw the adoption of COVID-19 vaccinations on a global scale. Vaccination for health care workers in Japan started in February 2021, and vaccinations for the elderly started in April 2021 [19]. The vaccination rate started picking up quickly in June 2021 and continued to rise until over 80% were fully vaccinated by the end of 2021 [20]. Meanwhile, vaccination in Indonesia started in January 2021, with health care workers as a priority, followed by the elderly and public officers, and finally for the general public [21]. Although the early vaccination campaign used the Coronavac and AstraZeneca vaccines, in August 2021, the Moderna and Pfizer vaccines started to be administered for vaccinations and boosters in the country. By the end of 2021, 46.7% of the population of Indonesia were fully vaccinated.

One key context behind vaccine hesitancy identified in both cultures is the role of side effects. In Japan, concerns about adverse side effects were arguably the main reason for vaccine hesitancy, alongside other factors like gender, living arrangements, economic status, and psychological issues [22]. Vaccine hesitancy was also found to be significantly more frequent in the younger generation than in the older generation [23]. Meanwhile, in Indonesia, concerns about vaccine safety, distrust toward the vaccine, and concerns about side effects were identified as a few common reasons for vaccine hesitancy [24]. As Twitter has been used to identify symptoms of COVID-19 [25], we proposed for this paper that it can also be utilized to examine side effects of COVID-19 vaccination. Moreover, by comparing side effect counts from Twitter with rates reported in phase 3 clinical trials of the Pfizer and Moderna vaccines, we can observe patterns in information dissemination on side effects that may be unique to Twitter (eg, are side effects overrepresented, appropriately, or underrepresented when mentioned on Twitter). These results may then determine the usefulness of Twitter for vaccine side effect monitoring or alternatively illustrate misinformation biases that are present on the platform.

Finally, we examined if there were differences in side effect reporting between the Pfizer and Moderna vaccines found in tweets by country. This is because publicly available research concerning vaccine (and maker-specific) side effects in both countries is still limited, and to our knowledge, only one study by Kitagawa et al [26] compared the side effects of the Moderna and Pfizer vaccines available in Japan through self-reported data. Analyses were conducted separately for Japan and Indonesia.

## Methods

### Data

#### Tweet Collection

To get a general sense of public opinions for the vaccination campaigns in Japan and Indonesia, tweets in Japanese (ja) and Indonesian (id; based on Twitter’s language filter) were collected for the whole of 2021, from January 1, 2021, to December 31, 2021 (UTC). The search query comprised keywords for vaccines and side effects and excluded retweets (see Table 1). Of all the vaccines used in both countries, we limited the query to Moderna and Pfizer because these 2 vaccines were used in both countries. Although AstraZeneca’s vaccine was also used in both countries, it was much less common in Japan and was represented by a variety of names in the public sphere, so relevant tweets were even more difficult to obtain.

All data were obtained using the Python Twarc library (version 2.8.3) for Academic Research Access in Twitter [27]. As limitations on tweet quota restricted access to vast amounts of Japanese tweets, we were unable to obtain tweets that mentioned “vaccine” only or “side effects” only. Consequently, the vaccine keywords used for Japanese and Indonesian tweet scraping included the vaccine type (“Pfizer” and “Moderna”), and the side effect keywords followed the symptoms described by the Centers for Disease Control and Prevention in English: tiredness, headache, muscle pain, chills, fever, and nausea [28]. Similarly, a list of symptoms was also available on the Indonesian government’s official webpage for effect after COVID-19 vaccination, which is abbreviated as *kipi* (in Indonesian: *kejadian ikutan pasca imunisasi*) [29]. Corresponding sources from the Japanese government also mentioned the same symptoms, excluding nausea but including diarrhea [30]. We decided to exclude diarrhea since it was not listed on the Indonesian source and the detailed prevalence is not available in Moderna public reports. However, we decided to retain nausea in this study because of the availability of the corresponding statistics, and it was also referenced by some research about vaccine side effects in Japan [26,31]. This list of symptoms was then translated into Japanese and Indonesian, with additional keywords added from synonyms (see Table 1).

**Table 1:** List of keywords related to COVID-19 vaccines.

Terms^a^			Keywords (delimited by commas)		
			Japanese		Indonesian
**Vaccine**					
	Vaccine-related	ファイザー, モデルナ		pfizer, moderna	
**Side effects**					
	Side effects	副反応		efek, kipi	
	Tiredness	疲労, 疲れ, 倦怠感, だるい, だるさ		lelah, capai, capek, pegal, lemas, letih	
	Headache	頭痛, 頭が痛い		pusing, sakit kepala	
	Muscle pain	筋肉痛		nyeri otot	
	Chills	寒気, 悪寒, さむけ		meriang, menggigil	
	Fever	熱, 高熱, 微熱, 発熱, 熱が高い, 熱があった, 熱がある, 熱が出た, 熱風邪		demam, panas	
	Nausea	悪心, 吐, 嘔吐, おう吐, 気分悪		mual	

^a^Translated into English.

#### Public Report Data

The comparison percentages listed in this paper were obtained from publicly available reports (press releases) published by Pfizer [32] and Moderna [33]. For Pfizer, these included data from participants aged 16 years to 55 years, and for Moderna, these included data from participants aged 18 years to 64 years. Both were collected for 7 days after the vaccination and classified as systemic adverse reactions.

### Preprocessing of Tweet Data

For tweets from both languages, the initial preprocessing steps were removing usernames and web links. Afterward, for Japanese tweets, we removed emojis and special characters (such as Japanese punctuation). Tweets were then tokenized using mecab-ipadic-NEologd [34-36], which reduced terms into their simplest forms to facilitate further analyses. All keyword filtering was done using full-width characters. For Indonesian tweets, all characters were set into lowercase, and non-ASCII characters were removed.

To assess the 2 vaccines separately, we filtered tweets to select tweets with the term Pfizer or Moderna only. Tweets mentioning both vaccines were removed and excluded from the analyses. Next, we defined user accounts with more than 10 tweets in our data as “high frequency users.” We removed these high frequency users to avoid having data biased by excessive tweet counts from the same individual. We then grouped tweets by user account, focusing on user-level analyses. If a user had more than one tweet, they were merged into a single sentence for the analyses. This was to reduce bias arising from the same individual tweeting their side effects multiple times over different tweets.

Following that, tweets with the term “Pfizer” were coded as 1, while tweets with the term “Moderna” were coded as 0. As mentioned earlier and considering the respective timelines for vaccinations, we excluded tweets mentioning both types (Pfizer and Moderna) and filtered them out at this stage. The resulting variable from this step served as the outcome variable for the logistic regression analysis.

The sample of filtered tweets is shown in Textbox 1. The tweet samples were paraphrased due to Twitter’s privacy policy. The Tweet ID for the data set processed in this study is available in Multimedia Appendix 1 (Japanese tweets) and Multimedia Appendix 2 (Indonesian tweets). In each filtered tweet, we applied word matching of the specified keywords to the merged tweets. If the word for side effect was present, then the column was marked as “1,” and if it was not, then it was marked as “0.” There were 7 predictor variables in total: effect, tiredness, headache, muscle pain, chills, fever, and nausea. The presence of each respective side effect was checked using exact word matching. For example, based on the Japanese tweet in Textbox 1, the columns for fever and headache would be marked as 1, while the rest of the side effects would be 0. Mentions of “pain” were not classified as “muscle pain,” so it was marked as 0.

**Examples of filtered tweets.**

Japanese tweet: こんばんは皆様。2回目のファイザーワクチンの接種後、夜から次の日にかけて、微熱と頭痛と接種部位の痛みを感じます。若い人の方が熱が出やすいと思われます。＃ファイザー＃コロナワクチン (Good evening everyone. After the second dose of Pfizer vaccine, I feel a slight fever, headache and pain at the vaccination site during the night and the next day. It seems that younger people are more prone to fever. #pfizer #coronavaccine)

Indonesian tweet: *efek dosis kedua vaksin moderna membuat aku menangis karena sakit demam, menggigil, badan terasa nyeri dan sakit kepala* (side effect of second dose of Moderna made me cry in pain with fever, chills, sore and headache)

### Logistic Regression Analysis

Logistic regression analysis is a statistical method to analyze associations with a binary outcome variable [37]. In this study, we conducted separate logistic regression models by country: Japan and Indonesia. The outcome variable was the vaccine type (Pfizer or Moderna), and the predictor variables were the identified side effects: side effect, tiredness, headache, muscle pain, chills, fever, and nausea (from the tweets). We then examined the likelihood of a specific side effect for each vaccine type by using the odds ratio obtained from the model parameter. A significance level of 5% was used to construct the confidence interval for the odds ratios. The model was evaluated using the Nagelkerke R^2^ [38]. All statistical analyses were conducted using SPSS version 28.0.1 (IBM Corp, Armonk, NY). All variables included in the analyses were binary.

### Ethical Considerations

This study did not require participants to be involved in any physical or mental intervention. As this research did not use personally identifiable information, it was exempt from institutional review board approval in accordance with the Ethical Guidelines for Medical and Health Research Involving Human Subjects stipulated by the Japanese national government.

## Results

### Comparisons With Public Report Data (Press Releases) From Clinical Trials

The final data set used in this research included 286,887 Japanese tweets from 214,165 users and 14,484 Indonesian tweets from 12,289 users. Table 2 shows the final tweet count after merging and the detailed breakdown for each side effect. For the final data set, the mean number of tweets per user for Japanese was less than the mean number of tweets per user for Indonesian, as shown in Table 2. However, since we aggregated tweets by user, we focused on user counts from merged tweets for subsequent analyses. The proportions of tweets about the Pfizer vaccine and Moderna vaccine in the Indonesian data set were 58.80% and 41.20%, respectively, with more tweets mentioning Moderna. For the Japanese data, 98.47% of the overall set of tweets mentioned Pfizer. The proportions of Pfizer and Moderna shots administered in Japan at the end of 2021 were 79.85% and 20.08%, respectively, and 0.08% for others (AstraZeneca) [39]. We were unable to access comparable statistics for Indonesia.

**Table 2:** Counts from the tweets.

		Japanese			Indonesian		
		Pfizer	Moderna	Total	Pfizer	Moderna	Total
Tweets, n		283,530	3357	286,887	5684	8800	14,484
Number of tweets per user, mean (SD)		1.344 (0.890)	1.028 (0.221)	1.339 (0.885)	1.122 (0.449)	1.218 (0.606)	1.178 (0.549)
Individual users (after merging tweets), n		210,899	3266	214,165	5063	7226	12,289
**Individual users who mentioned side effects from any vaccine, n**							
	Side effects	101,794	1224	103,018	1535	4628	6163
	Tiredness	39,724	406	40,130	375	356	731
	Headache	34,878	398	35,276	457	858	1315
	Muscle pain	25,167	339	25,506	9	37	46
	Chills	9361	143	9504	248	780	1028
	Fever	131,172	1993	133,165	2089	2386	4475
	Nausea	5334	49	5383	105	396	501

**Figure 1:**
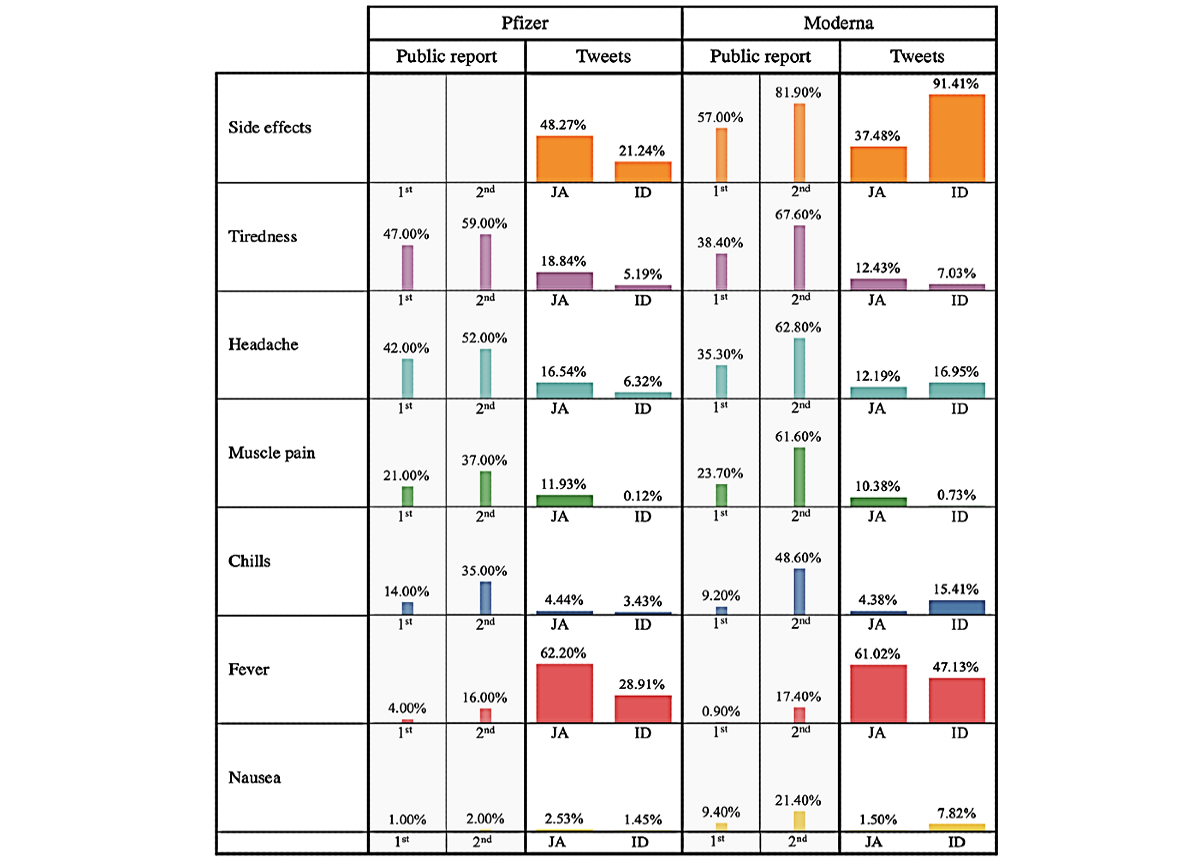
Percentage of side effects experienced after vaccination, as obtained from public reports by Pfizer and Moderna, compared with the percentages found in our Twitter sample. The overall percentage of general side effects was not mentioned in Pfizer’s report, and the percentage of side effects in tweets was calculated from the number of tweets for specific side effects divided by the total number of filtered tweets. 1st: first vaccine dose; 2nd: second vaccine dose; ID: Indonesian tweets; JA: Japanese tweets.

Figure 1 displays the side effects reported in press releases by Pfizer and Moderna, followed by the percentages of tweets observed in our study. The figure contains the side effects obtained from the word matching in tweets and the comparison with public report data to illustrate the ability of tweet data to capture the side effects. There appeared to be a difference between the percentage of side effects reported by press releases and that reported in tweet data and a slight difference between the percentages in Japanese and Indonesian tweets.

In the public reports, side effects were more frequently observed with the second dose than with the first dose. However, we lacked comparable information from the tweets to similarly differentiate side effects between the first dose and the second dose in our data set. Except for fever, we noticed that all side effects were reported more frequently in the public reports (higher percentages) than in the tweets. A radar graph showing the comparison of each side effect for the first dose and second dose in the public reports versus those obtained from the tweets is available in Multimedia Appendix 3, with the value plotted corresponding to the side effect counts in Table 2 and percentages from the values shown in Figure 1.

Looking into the tweet comparisons for Japanese and Indonesian tweets, for both Pfizer and Moderna, several side effects such as tiredness, muscle pain, and fever were reported at higher percentages in Japanese tweets than in Indonesian tweets. The percentages of headache, chills, and nausea were also different, with higher percentages in Indonesian tweets. Regardless of vaccine type, fever was by far the most reported side effect in tweets.

The percentages of all the side effects in Japanese tweets for the Pfizer vaccine were slightly higher than those for the Moderna vaccine, even when the total number of tweets for the 2 were notably different. On the other hand, in Indonesian tweets, the percentages of side effects with the Moderna vaccine were higher. We also noticed that the percentage reported for muscle pain was really small, which was probably caused by an inappropriate word choice used to represent this type of pain. For example, not many users may have been able to locate the exact part of the body from which the pain originated. A more general term “pain” may have been more suitable to represent this side effect than the specific term “muscle pain.”

### Logistic Regression Analysis of Vaccine Side Effects

We then compared Twitter mentions of side effects for the Pfizer and Moderna vaccines, with side effects as predictors and vaccine type (Pfizer or Moderna) as the outcome variable. Most of the predictor variables were significant (Table 3), suggesting that reported side effects differed significantly between Pfizer and Moderna vaccines. However, we note the high statistical power resulting from a large sample size may have affected the calculation of statistical tests and the respective *P* value. We first report results for the Japanese data, followed by the Indonesian data separately.

The Nagelkerke R^2^ for Japanese tweets was 1.2%. In interpreting the model, we found that the odds of the umbrella term “side effect” appearing in a tweet about the Pfizer vaccine was about 1.907 times more. For specific terms (ie, muscle pain, fever, headache, and nausea), the odds ratios were close to each other: They were 1.338, 1.357, 1.362, and 1.458 times more likely, respectively, to be mentioned in Pfizer tweets, suggesting that those terms were more frequent in tweets about the Pfizer vaccine than in tweets about the Moderna vaccine. However, only chills had a small odds ratio. This is different from past results reported by Kitagawa et al [26], who compared the prevalence of the side effects of both vaccine types through a questionnaire study conducted in Japan and found that users receiving the Moderna vaccine reported more side effects than those receiving the Pfizer vaccine.

Indonesian tweets showed a different result, as other than tiredness and fever, all other side effects appeared less likely to be mentioned in tweets about the Pfizer vaccine and more likely to be mentioned in tweets about the Moderna vaccine. The Nagelkerke R^2^ for the model for the Indonesian tweets was 17.4%. However, we noticed that tiredness appeared significantly more often in tweets about the Pfizer vaccine than in tweets about the Moderna vaccine. A closer look at the 95% CI for fever, which contained a value of 1, suggested that there may be little difference in those 2 side effects between the Pfizer and Moderna vaccines.

**Table 3:** Logistic regression analysis results for Japanese and Indonesian tweets.

Variable		Coefficient	Standard error	*P* value	Odds ratio	95% CI
**Japanese Tweets**						
	(Intercept)	3.551	0.043	<.001	34.831	—^a^
	Side effects	0.646	0.043	<.001	1.907	1.755-2.073
	Tiredness	0.489	0.054	<.001	1.631	1.468-1.814
	Headache	0.309	0.055	<.001	1.362	1.223-1.517
	Muscle pain	0.291	0.059	<.001	1.338	1.191-1.503
	Chills	–0.101	0.087	.25	0.904	0.762-1.072
	Fever	0.305	0.042	<.001	1.357	1.250-1.473
	Nausea	0.377	0.146	.01	1.458	1.095-1.941
**Indonesian Tweets**						
	(Intercept)	0.455	0.036	<.001	1.576	—
	Side effects	–1.461	0.042	<.001	0.232	0.214-0.252
	Tiredness	0.238	0.084	.004	1.269	1.077-1.495
	Headache	–0.294	0.068	<.001	0.745	0.653-0.851
	Muscle pain	–0.98	0.398	.01	0.375	0.172-0.818
	Chills	–1.029	0.080	<.001	0.358	0.306-0.418
	Fever	0.003	0.043	.94	1.003	0.922-1.092
	Nausea	–0.821	0.120	<.001	0.440	0.348-0.557

^a^Not applicable.

## Discussion

### Principal Findings

Our results highlight a large gap between expressions of side effects on Twitter and percentages reported in public press releases: For most of the side effects, we found that the percentages of Twitter users who reported them were far lower than the percentages reported in the public reports. Our first result is focused on the descriptive comparison of counts reported on Twitter and counts of observation in public press releases. Considering that several studies make use of symptom reporting on Twitter for epidemiological surveillance, our study shows that, at least for vaccine side effect surveillance, we may be at risk of overrepresenting “fever” relative to other (milder) side effects. Although our study did not explicitly examine the reasons behind this phenomenon, we speculate on a few possible explanations.

First, this could reflect a difference in how lay people and health professionals perceive and talk about vaccinations [40]. Our study may be relying too much on relatively specialized terminology (eg, muscle pain) that may not be the most salient term accessible to the broader lay population, at least in Japan and Indonesia. Hence, the difference in focus and word choice between lay people and professionals may result in different expressions used to describe side effects experienced after vaccination. Given that our study used more “professional” terms, our results for side effects like muscle pain or chills could thus have been an underrepresentation of available tweets.

Second, the higher reporting rates for fever could be due to its ease of measurement by lay members of the public. Thermometers are widely available and widely used, and there are general conventions (thresholds) for determining if a person has a fever. On the other hand, other side effects such as chills, headaches, and tiredness sometimes might not have clear, objective thresholds and measurement methods that are of common knowledge to the lay person. With the ambiguity and subjectivity around these side effects, Twitter users may hesitate to update their statuses, especially compared with fever, which comes with a clear and objective threshold. Accordingly, Twitter users who may experience more than one side effect may then decide to only report the clearer, more observable one.

Finally, another possible reason could be the age difference between people observed in the studies (public reports) and Twitter users who share their experiences in their tweets. In a survey conducted by Statista, close to 80% of Japanese respondents aged 20 years to 29 years reported using the microblogging and social networking service Twitter. Although this suggests that the penetration rate among Japanese youths was also on a high level, it was much less widely used by older age groups [41]. A past study also suggested that systemic incidence of side effects from the Pfizer vaccine was significantly higher in young participants than in older adults [31], a finding that was also previously extended to the Japanese context [42]. The rate of Twitter users in the country also shows that there are people who did not use Twitter, which means they will not share their side effects through tweets. As the incidence of the vaccine side effects is higher in older age groups while the Twitter penetration rate is lower, this might also influence the number of side effects that can possibly be found in Twitter.

Our findings from Japanese and Indonesian tweets were also different from reported (vaccine) side effects in English tweets in the United States, where soreness, fatigue, and headache were listed as the top 3 side effects for the Pfizer and Moderna vaccines [43]. One probable reason could be cultural differences in how people express themselves on Twitter, which might stem from their respective cultural background and habits. People in collectivist cultures (like Japan and Indonesia) may be less open and active on social media, as compared with individuals in individualistic cultures (like the United States) [44]. Accordingly, users in the United States could be reporting their symptoms with more detail and frequency on social media, whereas Japanese and Indonesian users may be “saving” their posts for worse side effects (ie, fever). In any case, we suggest that future studies on infodemiological surveillance of vaccine side effects may consider focusing primarily on fever-related keywords in these countries.

Regardless of interpretation, our study appears to suggest that “fever,” as a subjectively stronger side effect of vaccination, is discussed disproportionately more on Twitter in Japan and Indonesia. One possible consequence could be in the echo chamber effect on Twitter [45], which could contribute to vaccine hesitancy or other aversive behaviors. As an illustration, due to this disproportionate reporting, consider Marie, a Twitter user, who is currently considering vaccination. She may observe that many users on her Twitter feed discuss their experiences with fever as a side effect of vaccination, which could lead to a perceived overrepresentation of fever risks that may dissuade her from receiving the vaccine. In contrast, if tweets had discussed side effects in a more representative manner, Marie would have had an accurate representation of the risks and may not have been discouraged from vaccination for this reason. We note again that side effects were a strong reason for vaccine hesitancy in Japan [22] and postulate that this overrepresentation of strong side effects on Twitter may have had a role to play in contributing toward hesitancy, although follow-up research is needed to test this hypothesis.

### Limitations

Although we limited our investigation to only Moderna and Pfizer vaccines, these received relatively late approval in Indonesia, and we did not examine tweets on side effects from other vaccine makers (eg, Sinovac, AstraZeneca). Consequently, some of the discourse surrounding vaccines and their side effects was not captured in the earlier tweets. Second, some tweets also elaborated on side effects without mentioning the specific vaccine type received. Third, the search query for tweets was limited to the specified keywords (Table 1) and did not include other possible words not listed in the table, and we did not consider the positive or negative sentiment expressed by the tweets. Last, we focused only on tweets that mentioned one type of vaccine only and removed tweets mentioning both. Nevertheless, there was little chance of people getting both Pfizer and Moderna vaccinations administered in the observed period, and tweets mentioning both mainly referred to news articles and related discussions, not the actual side effects experienced by the public.

Finally, we did not control for negation in tweets. However, we sampled 100 tweets for each vaccine and language. Of all sampled tweets, we focused our observation on fever, which was the most frequent side effect found in our Twitter data set and found that only a minority of tweets contained negation. Based on a manual inspection of the sample, we found that, for Japanese tweets, negation (for fever) was observed in 15 of 63 tweets mentioning fever, or 23.80% of relevant tweets about the Pfizer vaccine. By doing the same process, we obtained 32.76% negation in Moderna tweets. Meanwhile, in Indonesian tweets, the negation for fever was 21.43% for the Pfizer vaccine and 16.67% for the Moderna vaccine. Negation is a difficult challenge in Twitter analyses, as there are many ways to express negation and may not necessarily be easily filtered out through designated negation words [46]. Although negation handling may improve the final results, it did not appear to hinder the utility of tweets in displaying consistent patterns as real-world (unfiltered) data in past research [47]. Considering that the observed percentage of negation for both vaccines in each language was similar in our random sample of tweets, we decided to retain all tweets for these analyses.

We also lacked the means to verify whether the tweets were from a personal or nonpersonal (eg, corporate) account and whether the said individual behind the account actually received vaccination and follow-up confirmations of reported side effects. Finally, we also lacked sufficient information about whether the side effects were from the first, second, or third dose of the vaccination, as we were limited to the side effects shared in the tweets by the users that matched the language filter of our Twitter API query without any deeper demographic or contextual information.

### Conclusions

We found that fever was the most prevalent side effect reported in Japanese and Indonesian tweets, and this may be a reflection of bias on social media toward reporting severe or measurable side effects (like fever). Furthermore, in examining side effects from different vaccine makers, we found that Twitter yielded inconsistent information from Japan and Indonesia, in that side effects were reported relatively more in tweets about the Pfizer vaccine in Japan but more in tweets about the Moderna vaccine in Indonesia. As such, given the inconsistencies and gaps in findings from Twitter and the vaccine press releases, we present cautious optimism that Twitter can prove useful for infodemiological surveillance for vaccine side effects that is best suited for detecting prevalences of fever symptoms in Japanese and Indonesian populations.

## Appendix

Multimedia Appendix 1 Tweet ID for Japanese tweets.

Multimedia Appendix 2 Tweet ID for Indonesian tweets.

Multimedia Appendix 3 Radar graph for Pfizer (A) and Moderna (B), showing comparisons of the percentages of each side effect.

## Abbreviations

JSPS: Japan Society for the Promotion of Science
JST: Japan Science and Technology Agency
mRNA: messenger RNA
SICORP: Strategic International Research Cooperative Program
