# Supplementary figures and images for: Monitoring Mentions of COVID-19 Vaccine Side Effects on Japanese and Indonesian Twitter: Infodemiological Study
Source: JMIR Infodemiology. 2022 Oct 4;2(2):e39504. doi: 10.2196/39504 (PMC9578292; doi:10.2196/39504)

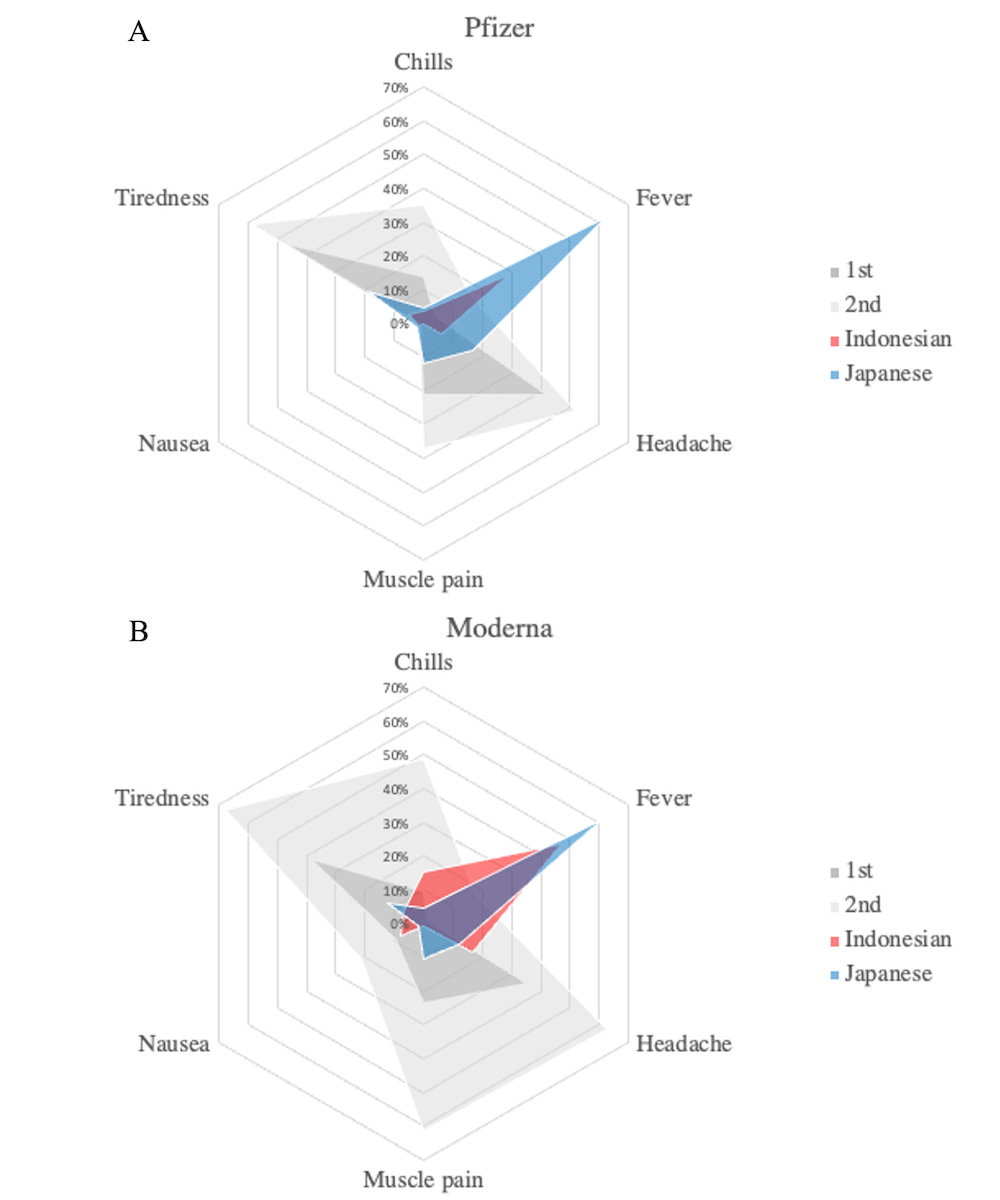

Supplement: Multimedia Appendix 3 [file infodemiology_v2i2e39504_app3.png]
